# Down-Regulation of CDH1 Is Associated with Expression of SNAI1 in Colorectal Adenomas

**DOI:** 10.1371/journal.pone.0046665

**Published:** 2012-09-28

**Authors:** Feride Kroepil, Georg Fluegen, Zaurbek Totikov, Stephan E. Baldus, Christian Vay, Matthias Schauer, Stefan A. Topp, Jan Schulte am Esch, Wolfram T. Knoefel, Nikolas H. Stoecklein

**Affiliations:** 1 Department of General-, Visceral- and Pediatric Surgery, University Hospital Düsseldorf, Düsseldorf, Germany; 2 Institute for Pathology, University Hospital Düsseldorf, Düsseldorf, Germany; University of Munich, Germany

## Abstract

**Introduction:**

Down-regulation of E-cadherin (CDH1) and epithelial-mesenchymal transition (EMT) are considered critical events for invasion and metastasis of colorectal carcinoma. Here we tested whether the important regulators of E-cadherin expression SNAI1 and TWIST1 are already detectable in human colorectal adenomas.

**Methods:**

RNA was extracted from a set of randomly selected formalin-fixed and paraffin-embedded (FFPE) colorectal adenomas (n = 41) and normal colon mucosa (n = 10). Subsequently mRNA expression of CDH1, CDH2, SNAI1 and TWIST1 was analysed by quantitative RT-PCR analysis. CDH1 as well as SNAI1 protein expression were assessed by immunohistochemistry (IHC).

**Results:**

SNAI1 mRNA was expressed in 78% (n = 32/41), TWIST1 mRNA in 41% (n = 17/41) and CDH2 mRNA in 41% (n = 17/41) of the colorectal adenoma tissue, while normal colon mucosa was negative for these transcription factors. We found a significant correlation between reduced CDH1 and the presence of SNAI1 mRNA expression and for combined SNAI1 and TWIST1 mRNA expression, respectively. A correlation between CDH2 mRNA expression and reduced CDH1 expression was not observed. We confirmed the relationship between SNAI1 expression and reduced E-cadherin expression on the protein level via IHC.

**Conclusion:**

Our data show that SNAI1 and Twist1 are already expressed in benign precursor lesions of colorectal cancer and that SNAI1 expression was significantly correlated with lower expression of CDH1. Whether these findings reflect true EMT and/or are a sign of a more aggressive biology need to be investigated in further studies.

## Introduction

Epithelial-mesenchymal transition (EMT) denotes a process in which cells change their phenotype between epithelial and mesenchymal states. This phenotypic change involves complex molecular and cellular programs by which epithelial cells can dispose of their differentiated characteristics, including cell-cell adhesion, planar and apical-basal polarity, lack of motility and gain instead mesenchymal features such as motility, invasiveness and increased apoptotic resistance [1]. The reversible EMT process is crucial in embryonic development for correct implantation of the embryo and later, to control epithelial plasticity during gastrulation and during organogenesis [2], [3]. In differentiated somatic cells the tightly controlled EMT programs are normally shut off. However, as physiologic response to injury, strictly coordinated processes similar to EMT can occur with limited duration [3]. E.g. adult keratinocytes can express the EMT-inducing transcription factor SNAI2 (Slug) after injury at the wound edges for enhanced migratory ability and effective wound re-epithelialization [4].

Ostensibly, the ‘uncontrolled’ reactivation of such EMT programs occurs frequently in cancer cells [3], [5]. In the context of cancer, EMT is mainly discussed as promoter of metastasis, enabling motility and invasion of epithelial cancer cells, and their dissemination to distant organs [2]. EMT programs also appear to confer stem cell properties, resistance to apoptosis and senescence, act on immunosuppressive mechanisms, and enhance resistance against systemic cancer drugs [3], [6]. All of these pleiotropic oncogenic effects seem to occur late in cancer progression and are believed to foster the switch between the benign and the malignant, systemic disease. While a relative coherent picture exists about the onset and timing of the physiological EMT program activation during embryonic development [3], the onset is less clear in cancer. Considering the attributed role of EMT in cancer one would not expect aberrant activation in benign tumors. However, this has not yet been investigated in detail. To address this issue, we tested a series of randomly selected benign colorectal adenomas for the expression of the EMT inducers SNAI1 and TWIST1, as well as the mesenchymal marker N-cadherin. Among the many known transcription factors regulating EMT, we focused on SNAI1 and TWIST1 because (i) both are considered as master regulators of EMT and are as such examples for direct (Snail) and indirect (Twist) suppressors of E-Cadherin (CDH1) [3], (ii) both are considered to be important for metastasis in several cancer types [1], [3], [6], and (iii) the aberrant expression of both is frequently reported in colorectal cancer (CRC) [7], [8], [9], [10], [11], [12], [13]. Importantly, their aberrant expression in CRC was found to be associated with poor prognosis and shortened relapse-free survival [7], [8], [10], [11]. As an early event in EMT, cells undergo a cadherin switch, expressing N-cadherin (CDH2) instead of E-cadherin (CDH1). This switch has been proven to be essential for gastrulation and mesoderm formation [14]. In cancer, N-cadherin expression has been associated with increased motility and invasiveness [15], [16], [17].

In order to investigate, whether the EMT “master regulators” SNAI1 and TWIST1 and the mesenchymal marker CDH2 are already expressed in colorectal adenomas, we assessed their expression in formalin fixed and paraffin embedded (FFPE) tissues and used previously published primers and probes for a quantitative RT-PCR assay (qPCR) that were shown to work well in FFPE material [18]. Furthermore, we tested the association between the expression of CDH1 and SNAI1/TWIST1 expression and validated our transcriptional data on protein expression level.

## Materials and Methods

### Patients

Colorectal adenoma specimens obtained in the period 2002 to 2007 were retrieved from the files of the Department of Pathology (University Hospital Düsseldorf). All patients suffering from known hereditary colorectal cancer syndromes were excluded. A total of 41 benign colorectal adenoma specimens of 35 patients were randomly selected. In addition, normal colonic mucosa (n = 10) and colorectal cancer tissue (n = 10) from the same period were selected. This study was approved by the Ethics Committee of the Medical Faculty of the Heinrich-Heine University Düsseldorf, they waived the need for written informed consent for using the patients' material, as it was analysed anonymously. This is also in accordance to the recommendation of the German Central Ethics Committee from 2003.

### Clinicopathological characteristics

Our series consisted of 63% (n = 22) male and 37% (n = 13) female patients; the average age at the moment of resection was 68 years. All adenomas were graded and classified according to histologic type and degree of intraepithelial neoplasia by experienced pathologists. **Table 1** shows a complete list of the adenomas and the corresponding clinicopathological characteristics.

**Table 1:** Characteristics of patients included in this study.

Adenoma	Case	Sex	Age	Size	Histology	Dysplasia
1	1	M	78	0,7	tubular	low grade
2	2	M	51	0,7	tubular	low grade
3	3	F	76	1,5	tubulovillous	low grade
4	4	M	76	0,2	tubular	low grade
5	5	F	86	0,2	tubular	low grade
6	6	M	74	2,0	tubulovillous	low grade
7	7	F	41	0,2	tubular	low grade
8	8	M	62	1,4	tubulovillous	low grade
9	9	M	73	3,0	tubulovillous	low grade
10	10	M	72	0,5	tubulovillous	low grade
11	10	M	72	0,5	tubular	low grade
12	11	F	85	2,0	tubulovillous	low grade
13	12	M	68	0,5	tubular	low grade
14	13	M	60	3,0	tubular	low grade
15	14	M	79	0,5	tubular	low grade
16	14	M	79	1,0	tubular	low grade
17	14	M	79	1,5	tubular	low grade
18	14	M	79	5,0	tubular	high grade
19	15	F	71	4,2	tubulovillous	low grade
20	16	M	54	0,2	tubular	low grade
21	17	M	68	0,5	tubular	low grade
22	18	F	58	0,5	tubular	low grade
23	19	F	64	0,5	tubular	low grade
24	20	M	60	0,9	tubulovillous	low grade
25	21	M	51	1,5	tubulovillous	low grade
26	22	M	68	0,5	tubular	low grade
27	23	M	63	1,5	tubulovillous	low grade
28	23	M	63	3,0	tubulovillous	high grade
29	24	F	65	0,7	tubular	low grade
30	24	F	65	1,1	tubular	high grade
31	25	F	83	1,0	tubular	low grade
32	26	M	78	1,5	tubulovillous	low grade
33	27	M	97	4,5	tubular	high grade
34	28	M	63	8,0	tubulovillous	high grade
35	29	F	72	1,2	tubulovillous	high grade
36	30	M	60	1,0	tubular	high grade
37	31	F	75	2,3	tubular	high grade
38	32	F	75	0,5	tubular	high grade
39	33	M	75	1,7	tubular	high grade
40	34	M	54	3,0	tubular	high grade
41	35	F	46	0,6	tubular	high grade

Size: greatest possible diameter in centimetres.

### Quantitative RT-PCR

Total RNA was extracted from 4 µm serial sections of formalin-fixed paraffin-embedded (FFPE) specimens. Paraffin was removed by extracting two times with xylene for 5 minutes followed by rehydration through a graded ethanol series (100, 95, 70% ethanol). After the final 70% ethanol wash and subsequent rinsing in phosphate buffered saline (PBS, pH 7.4), the specimens were immersed in 3% glycerol for 30 seconds. Microdissection was carried out using sterile equipment and samples were transferred in a sterile 1.5-ml tube containing 1000 µl TRIzol reagent (Invitrogen, UK). Lysis was carried out at room temperature for at least 10 minutes or until the tissue was completely solubilized. The RNA was purified by chloroform extraction, followed by precipitation with an equal volume of isopropanol at room temperature. The RNA pellet was washed once with 75% ethanol, air-dried, and re-suspended in 30 µl of RNase-free water. The total RNA and a human reference RNA (1 ng/µl) (Clon tech, Canada) were reverse-transcribed in a total volume of 20 µL consisting of: 2 µl random hexamere primer, 20 U Protector RNase-Inhibitor, 2 µl dNTPs (10 mmol/L), 0.5 µl reverse transcriptase and 11 µl total RNA (containing a maximum of 2 µg RNA) in 5x RT-buffer (all: Roche Diagnostics, Germany). The reaction mixture was incubated at 25°C for 10 min followed by 30 min at 55°C. The enzyme inactivation step was carried out for 5 min at 85°C. The cDNA was stored at −20°C until use.

Quantitative RT-PCR analyses for SNAI1, TWIST1, CDH1, CDH2 and GAPDH were performed using the Dyad Disciple Chromo 4 instrument and software (BioRad, Germany). Intron-spanning primers and probes for the TaqMan system were selected from literature [18] and cross-checked using the BLAST library (http://blast.ncbi.nlm.nih.gov/Blast.cgi). The sequence of the PCR primer pairs and probes are shown in **Table 2**. All primers and probes were purchased from eurofins MWG, Germany. Quantitiative RT-PCR was performed in a total reaction volume of 25 µl containing 12.5 µl iQ Supermix mastermix® (BioRad, Germany), 1.25 µl (30 pmol) primermix and 11.25 µl cDNA, or water as control. Thermal cycling conditions included 2 min at 50°C to allow for cleavage of cDNA double-strands and 10 min at 95°C to activate the Taq polymerase, followed by 45 cycles at 95°C for 15 sec and 60°C for 1 minute.

**Table 2:** List of primers and probes used used for qRT-PCR.

Transcript		Sequence
GAPDH	Reverse	5′-GCC ATC ACG CCA CAG TTT C-3′
	Forward	5′-CGT GGA AGG ATC CAT GAC CA-3′
	Probe	5′-CAG AAG ACT GTG GAT GGC CCC TCC-3′
CDH1	Reverse	5′-GCA GAA CTG TCC CTG TCC CAG-3′
	Forward	5′-GAA CAG CAC GTA CAC AGC CCT-3′
	Probe	5′-ATC ATA GCT ACA GAC AAT GGT TCT CCA GTT GCT-3′
SNAI1	Reverse	5′-GTG GGA TGG CTG CCA GC-3′
	Forward	5′-TGC AGG ACT CTA ATC CAA GTT TAC-3′
	Probe	5′-TCC AGC AGC CCT ACA CCA GGC C-3′
TWIST1	Reverse	5′-TGT CCA TTT TCT CCT TCT CTG GA-3′
	Forward	5′-TGT CCG CGT CCC ACT AGC-3′
	Probe	5′-TCA GCA GGG CCG GAG ACC TAG ATG T-3′
CDH2	Reverse	5′ - TCG ATT GGT TTG ACC ACG G - 3′
	Forward	5′ - GAC GGT TCG CCA TCC AGA C - 3′
	Probe	5′ - ACC CAA ACA GCA ACG ACG GGT TAG TC - 3′

Relative expression levels of target sequences were determined by the comparative C_t_ method (2^−ΔΔCt^) using GAPDH as housekeeping gene and a human reference RNA as external calibrator. We normalized the resultant C_t_-values to the housekeeping gene, thus creating ΔC_t_-values (ΔC_t_ = C_t(target)_−C_t(housekeeping)_). In a next step, we calculated the ΔΔC_t_-values, using the equation ΔΔC_t_ = ΔC_t(target)_−ΔC_t(calibrator)_. ΔC_t(target)_ is the C_t_-value of any target gene normalized to the endogenous housekeeping gene and ΔC_t(calibrator)_ is the C_t_-value of the same gene in the human reference RNA also normalized to the endogenous housekeeping gene. For all probes and primer pairs we determined the efficiency by the standard curve method. Since the efficiencies of our probes and primer pairs were approximately equal, we could use the comparative equation 2^−ΔΔCt^ to calculate the relative amount of target gene [19]. The resultant values were used for further statistical evaluation.

### Immunohistochemistry

We used tissue from the same blocks as for the qRT-PCR, except for one case in which no further tissue was available. For immunostaining, 4 µm serial sections were stained as described in [20], [21]. **Table 3** summarizes the antibodies used in this study. Negative controls were constructed by using IgG1 (mouse monoclonal, MOPC-21, 2 µg/ml, Sigma-Aldrich, USA) and X0903 (rabbit immunoglobulin fraction, 1 µg/ml, Dako Cytomation, USA) as primary antibodies.

**Table 3:** List of primary antibodies used for immunohistochemistry.

Antigen	Antibody	Concentration	Animal source
E-Cadherin	NCH-38, DAKO	2 µg/ml	Mouse
Snail1	Ab17732, AbCam	1 µg/ml	Rabbit
Isotype control	MOPC-21, Sigma	2 µg/ml	Mouse
Isotype control	X0903, DAKO	1 µg/ml	Rabbit

Two independent observers examined the sections. Normal colonic mucosa adjacent to the adenomas was used as internal control. For E-cadherin, we used a scoring system that included an evaluation of both the staining intensity and the percentage of stained cells similar to Blechschmidt et al. [22]. Staining intensity was classified from 0 to 3+: 0 (no staining), 1+ (weak staining), 2+ (moderate staining) or 3+ (strong staining). Strong E-cadherin staining in >20% of cells was regarded as preserved, strong staining in <20% as down regulated. In the evaluation of Snail1 staining, only nuclear staining was considered. Adenomas with detectable nuclear Snail1 staining were considered positive, while adenomas with no detectable nuclear Snail1 staining were considered negative.

### Statistical analysis

Statistical analysis was performed with the SPSS software (SPSS Standard version 17.0.0, SPSS Inc., Chicago, IL). Significance of differences between groups with a nonparametric data distribution was analyzed with the Mann–Whitney U test for two independent groups. The threshold for statistical significance was chosen at p<0.05.

## Results

### Quantitative gene expression analysis

**Figure 1:**
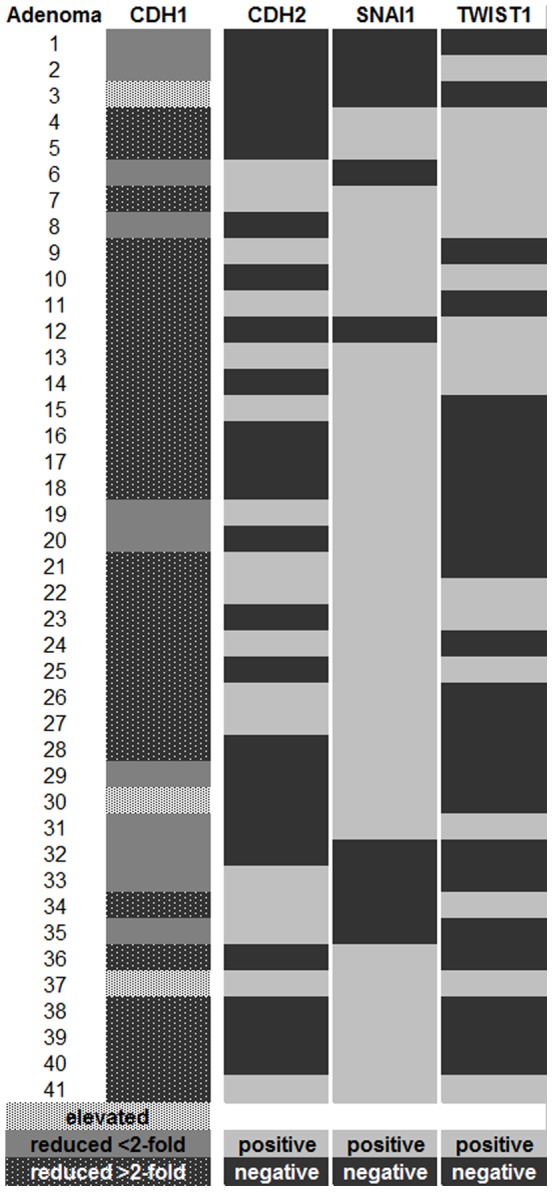
Expression profile of colorectal adenomas in the qRT-PCR. CDH1: up- and downregulation compared to normal colonic mucosa.

We performed a quantitative RT-PCR assay to analyze the mRNA expression of SNAI1 (Snail1), TWIST1 (Twist1), CDH1 (E-cadherin) and CDH2 (N-cadherin) in FFPE tissue samples of colorectal adenomas (n = 41), colorectal cancer (n = 10) and normal colon mucosa (n = 10). In normal colonic mucosa, SNAI1, TWIST1 and CDH2 mRNA could not be detected at all. We could detect SNAI1 mRNA expression in 32 (78%) and TWIST1 mRNA expression in 17 (42%) of 41 colorectal adenomas. 13 out of 41 (32%) colorectal adenomas expressed both SNAI1 and TWIST1 mRNA at the same time. In 17 of the 41 (42%) colorectal adenomas, we detected CDH2 mRNA **(** **Fig. 1** **)**.

**Figure 2:**
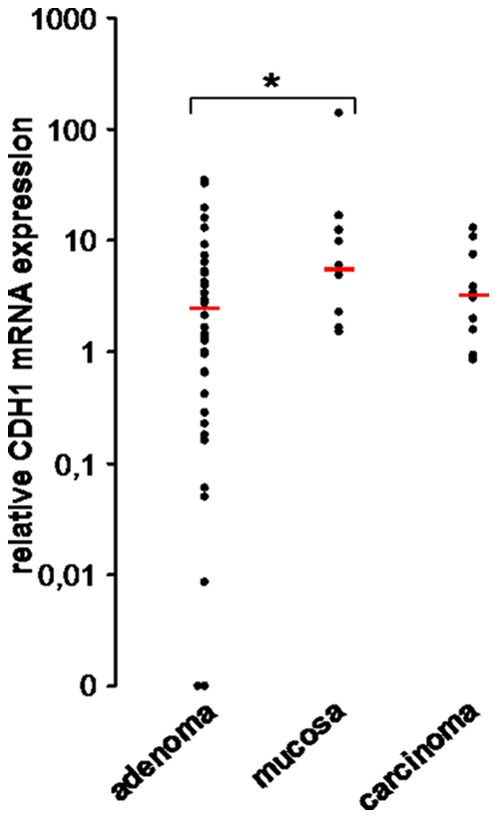
CDH1 mRNA expression in colorectal adenoma, normal colonic mucosa and colorectal carcinoma. Y-axis: relative amount of CDH1 mRNA on a log-scale; x-axis: sample tissue. *p = 0.035. Bars indicate the median value.

CDH1 mRNA expression was detected in 39 of 41 (95%) colorectal adenoma samples, in all 10 (100%) colorectal cancer samples and in all 10 (100%) normal colonic mucosa samples. CDH1 expression was significantly reduced in colorectal adenomas compared to normal colonic mucosa (p = 0.035, Mann-Whitney-U test). As expected, expression of CDH1 mRNA was clearly decreased in colorectal cancer compared to the expression in normal colonic mucosa. Due to the small number of samples, this correlation did not reach significance (p = 0.199, Mann-Whitney-U test). The amount of CDH1 mRNA did not differ significantly between colorectal adenoma and colorectal cancer **(** **Fig. 2** **)**.

**Figure 3:**
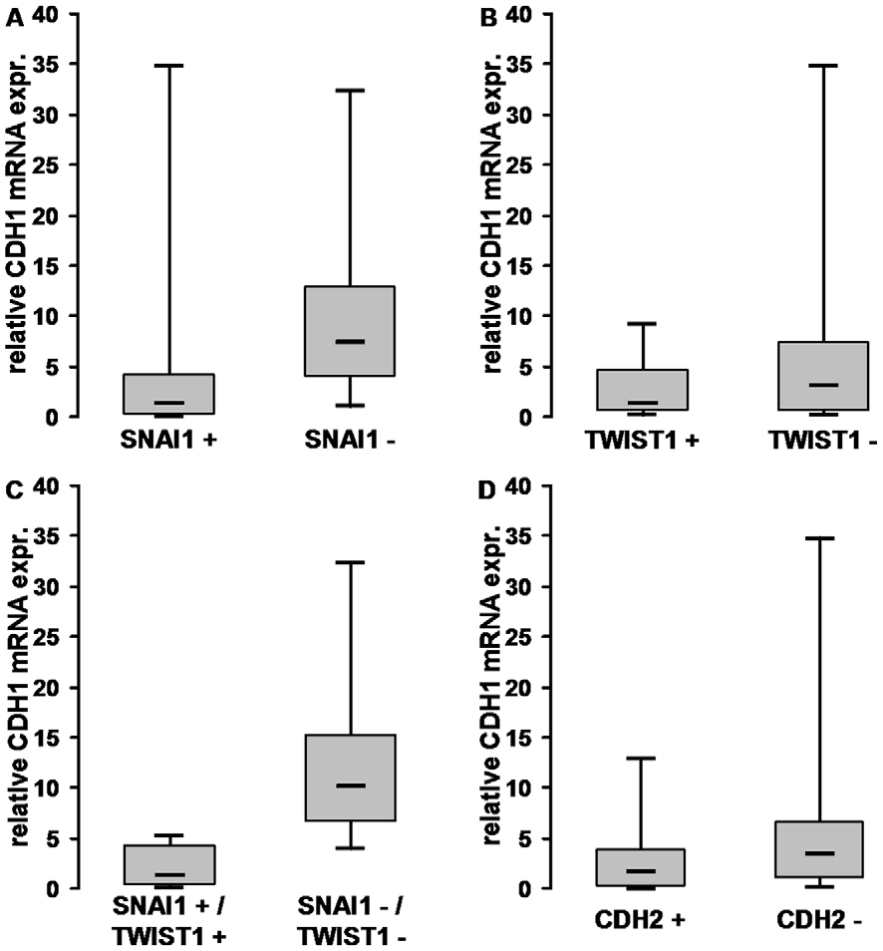
Expression of CDH1 mRNA in correlation to SNAI1, TWIST1 and CDH2 mRNA occurrence. See Methods for details on qRT-PCR and quantification. Y-axis: relative amount of CDH1 mRNA on a metric scale; X-axis: adenomas positive or negative for target transcript. Boxed regions enclose 25th to 75th percentiles, with the horizontal line indicating the median. Whiskers include 5th to 95th percentiles. **A:** The amount of CDH1 mRNA was significantly lower in SNAI1 positive adenomas compared to SNAI1 negative ones (p = 0.004). **B:** TWIST1 positive adenomas had a reduced amount of CDH1 mRNA, but the difference to TWIST1 negative adenomas did not reach significance (p = 0.29). **C:** Co-expression of SNAI1 and TWIST1 showed a highly significant reduction in CDH1 mRNA (p = 0.003). **D:** Adenomas with expression of CDH2 mRNA did not show any significant difference in the amount of CDH1 mRNA compared to adenomas without CDH2 mRNA (p = 0.24).

Next, we tested whether the expression of SNAI1 and TWIST1 mRNA correlated with the expression of CDH1 mRNA. When comparing the amount of CDH1 mRNA in colorectal adenomas with or without SNAI1 expression, we found a significant difference. In the 32 colorectal adenomas, which were positive for SNAI1 mRNA, the amount of CDH1 mRNA was significantly lower (p = 0.004, Mann-Whitney-U test), compared to the amount in colorectal adenomas without SNAI1 mRNA expression **(** **Fig. 3A** **)**. Colorectal adenomas positive for TWIST1 mRNA also showed a lower amount of CDH1 mRNA - the correlation was not significant (p = 0.29, Mann-Whitney-U test) **(** **Fig. 3B** **)**. In the case of co-expression of both SNAI1 and TWIST1 mRNA, the amount of CDH1 mRNA was still significantly reduced (p = 0.003, Mann-Whitney-U) **(** **Fig. 3C** **)**.

**Figure 4:**
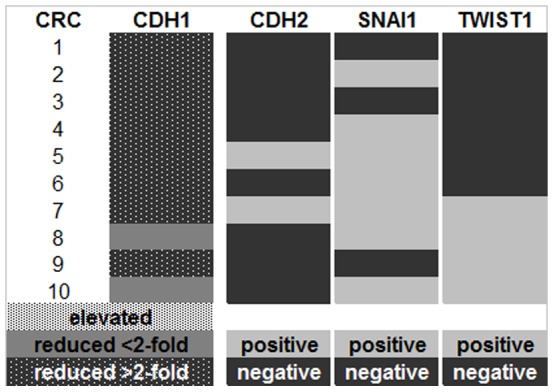
Expression profile of carcinomas in the qRT-PCR. CDH1: up- and downregulation compared to normal colonic mucosa.

Even though it was often proposed that N- and E-cadherin have contrary functions in cancer progression [15], [17], [23], we could not find a significant inverse correlation between CDH1 and CDH2 mRNA expression in colorectal adenomas or carcinomas. All 17 of the CDH2 positive colorectal adenomas were also positive for CDH1 mRNA. Of the 10 colorectal carcinomas, two expressed CDH2 mRNA and both showed reduced, but detectable, levels of CDH1 mRNA **(** **Fig. 4** **)**. The amount of CDH1 mRNA did not differ significantly when comparing CDH2 positive and CDH2 negative colorectal adenomas (p = 0.24, Mann Whitney-U test) or carcinomas (p = 1.0, Mann Whitney-U test), respectively **(** **Fig. 3D** **)**.

We did not detect any correlation between the genes of interest and the histologic type (tubular or tubulovillous adenoma), the grading (low or high grade) or the size (<1 cm or ≥1 cm) of the adenomas. Also there was no correlation to the age at diagnosis (<68 years or ≥68 years) or the gender of the patients.

### Correlation to immunohistochemical staining

**Figure 5:**
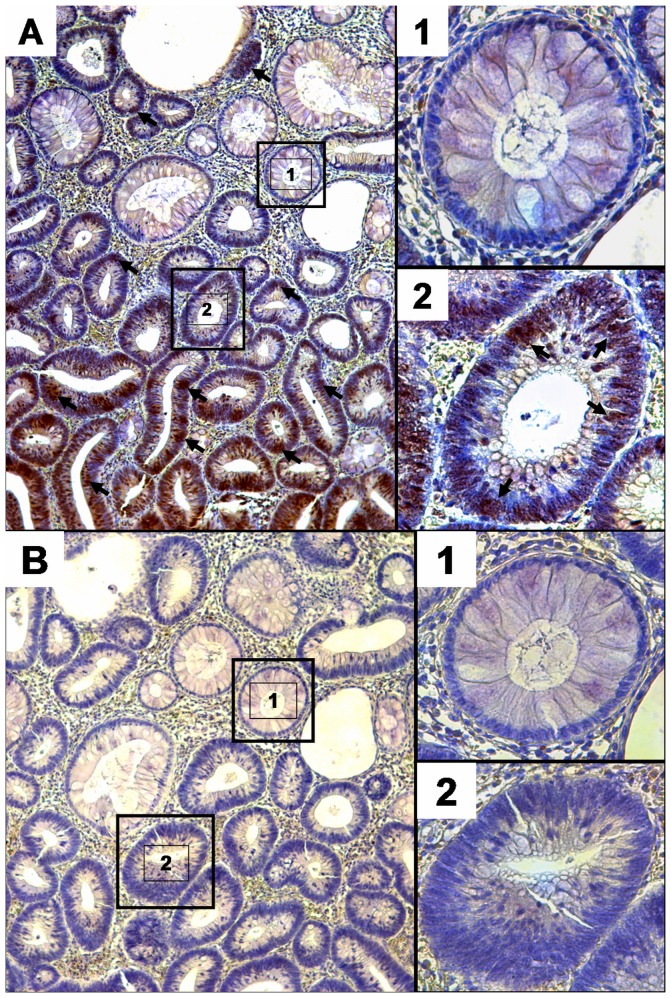
Snail1 expression in normal colonic mucosa and colorectal adenoma. Expression of Snail1 was determined as indicated in Methods using Ab17732 antibody as positive and X0903 antibody as negative control. Panels A and B show corresponding areas of a colorectal adenoma. Panel A corresponds to Snail1 staining (arrows = Snail1 positive cells), while panel B shows the negative control. A1: adenomatous tissue negative for Snail1 staining. A2: colorectal adenoma tissue positive for nuclear Snail1 staining Panels B1 and B2: no positive reaction in negative control.

**Figure 6:**
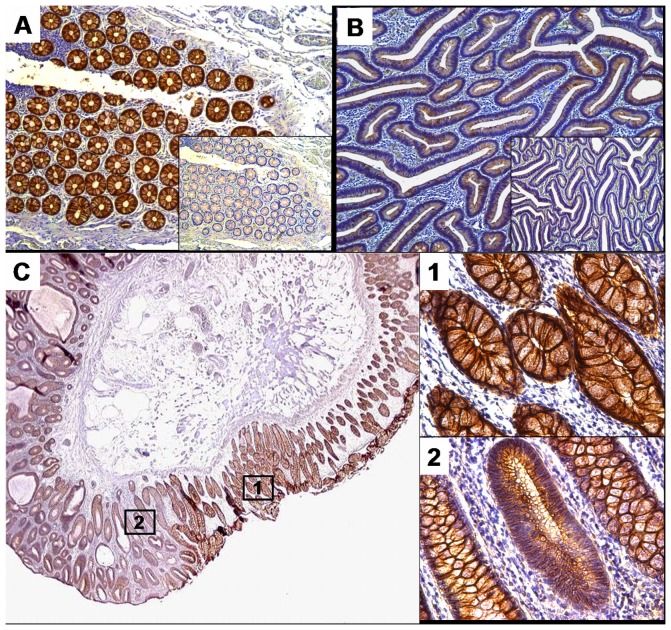
E-cadherin expression in normal colonic mucosa and colorectal adenoma. Expression of E-cadherin was determined as indicated in Methods using NCH-38 antibody and MOPC-21 as isotype control. Panels A and B show normal colonic mucosa and colorectal adenoma tissue (respectively). Note the difference in E-cadherin expression. The inlays in panels A and B correspond to the negative control of the same sample. Panel C shows an overview of a colorectal adenoma with adjacent normal colonic mucosa. C1 and C2 correspond to the indicated areas in panel C and show normal colonic mucosa with normal E-cadherin staining (C1) and colorectal adenoma with reduced E-cadherin staining (C2).

To correlate our qRT-PCR results on the protein level, we performed an immunohistochemical analysis of Snail1 and E-cadherin protein in 40 of the colorectal adenomas. For Snail1, only detectable nuclear staining was considered positive **(** **Fig. 5** **)**. For E-cadherin, only strong membranous staining in >20% of tumour cells was considered preserved **(** **Fig. 6** **)**. E-cadherin immunoreactivity was preserved in 17 (43%) and reduced in 23 (57%) of 40 colorectal adenomas. Snail1 nuclear staining was positive in 27 (68%) and negative in 13 (32%) of 40 colorectal adenomas.

**Figure 7:**
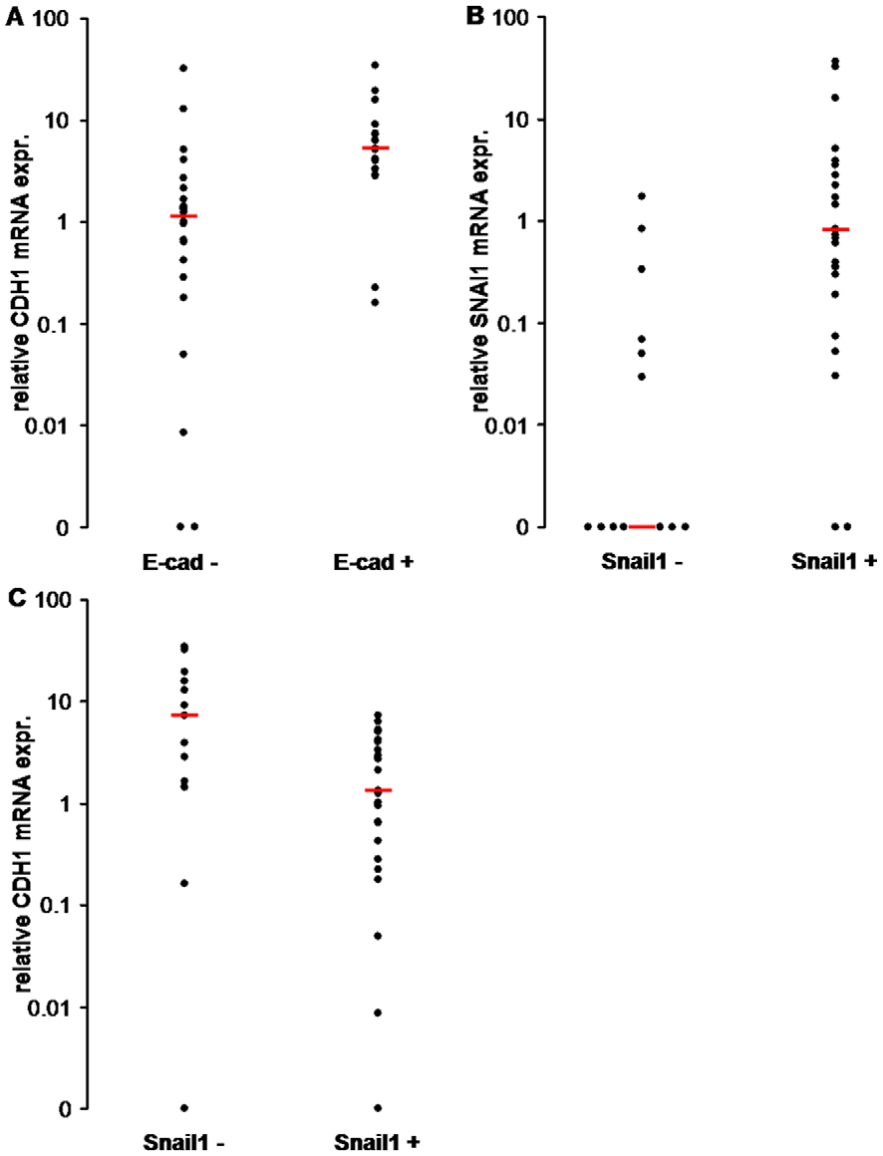
Correlation of qRT-PCR and immunohistochemistry. Y-axis: relative amount of target mRNA in the qRT-PCR on a log.scale; X-axis: positive or negative immunostaining for target protein. Bars indicate median value. **A:** Adenomas positive for E-Cadherin protein in the immunohistochemistry had a significantly lower expression of CDH1 mRNA in the qRT-PCR, compared to immunohistochemically negative ones (p = 0.003). **B:** Adenomas with positive nuclear Snail1 staining in the immunohistochemistry had significantly higher amounts of SNAI1 mRNA in the qRT-PCR, compared to adenomas negative for Snail1 protein in the immunohistochemistry (p = 0.001). **C:** Adenomas positive for nuclear Snail1 staining in the immunohistochemistry showed significantly lower amounts of CDH1 mRNA in the qRT-PCR, compared to those negative for Snail1 protein in the immunohistochemistry (p = 0.02).

The Snail1 immunohistochemistry correlated significantly with the level of CDH1 mRNA (p = 0.02, Mann-Whitney-U test). Adenomas with positive Snail1 nuclear immunostaining had a lower level of CDH1 mRNA and with absent nuclear Snail1 staining showed higher levels of CDH1 mRNA **(Fig. 7C)**. This correlation further indicated an influence of SNAI1 on the expression of E-Cadherin in colorectal adenomas.

The colorectal adenomas with preserved E-cadherin staining showed a significantly higher amount of CDH1 mRNA in the qRT-PCR, compared with colorectal adenomas with reduced E-cadherin immunoreactivity (p = 0.003, Mann-Whitney-U test) **(Fig. 7A)**. We found the same significant correlation between Snail1 positive colorectal adenomas and a high amount of SNAI1 mRNA, as well as between Snail1 negative colorectal adenomas and low amounts of SNAI1 mRNA (p = 0.001, Mann-Whitney-U test) **(Fig. 7B)**. These findings confirm that increased levels of CDH1 and SNAI1 mRNA were consistent with higher protein expression in the investigated colorectal adenomas.

**Figure 8:**
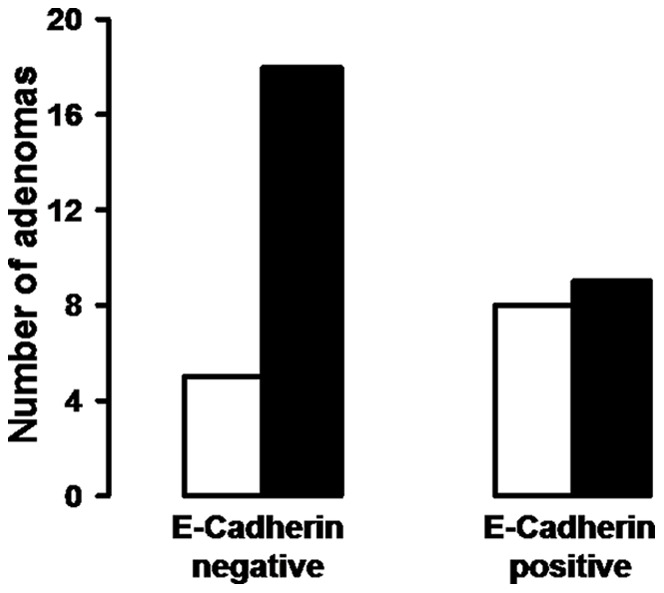
Correlation of Snail1 and E-cadherin protein expression in the immunochemistry. Y-axis: number of adenomas; white column = Snail1 negative; black column = Snail1 positive (p = 0.095).

On the transcriptional level, we observed a significant correlation between SNAI1/Snail1 expression and CDH1/E-cadherin loss in colorectal adenomas **(** **Fig. 3A** **, 7C)**. This observation is in agreement with the role of SNAI1 as transcriptional repressor of E-cadherin protein. But no correlation between TWIST1 and CDH1 mRNA was noted. However, when co-expressed with SNAI1, there were slightly lower levels of CDH1 noted compared to SNAI1 alone **(** **Fig. 3C** **)**. When we compared the expression of Snail1 and E-cadherin using immunohistochemistry, we could observe a trend towards a correlation between a nuclear Snail1 staining and lower E-cadherin protein expression (p = 0.095, Mann-Whitney-U test) **(** **Fig. 8** **)**.

## Discussion

It has been clearly shown in a variety of model systems that cancer cells use EMT to down-regulate their cell-cell contacts and to become motile and invasive [5]. Many authors regard EMT as a major mechanism enabling metastasis and initiating the transition between benign and malignant disease. Consequently, one would not expect frequent expression of EMT master regulators in benign tumors. Analysing an unselected cohort of colorectal adenomas, we were therefore surprised by the relatively high frequency of SNAI1 and TWIST1 mRNA expression, which was quite similar to the published expression rates in CRC tissue. The previously reported expression rates in CRC were 50–78% for SNAI1 [9] and 40%–80% for TWIST1 [8], [10], [13], respectively. In contrast, and as expected, SNAI1 and TWIST1 mRNAs were not detected in morphologically normal colon mucosa by our qRT-PCR assay. Strikingly, mRNA expression of SNAI1 was significantly correlated with decreased levels of CDH1 mRNA in colorectal adenomas, suggesting an “active” CDH1 suppression by the transcription factor SNAI1. Although the correlation between TWIST1 expression and CDH1 levels did not reach statistical significance, a lower mean CDH1 level was noted for TWIST1 positive adenomas.

For our qPCR-assay we used primers and probes published by Rosivatz et al. [18], which were tested for FFPE samples. As in our current study on colorectal adenoma tissue, they observed in diffuse gastric cancer that increased SNAI1 mRNA expression was associated with down-regulation of CDH1 mRNA [18]. However, when they applied their qPCR assay to 16 CRC they did not observe TWIST1 expression and SNAI1 was only rarely detected in 31 investigated CRC tissues [23]. A possible explanation for this discrepancy to our data might be a higher sensitivity of the qPCR assay used by us due to the different chemistry and set-up of the assay. However, the expression frequencies of SNAI1 and TWIST1 observed in our study are in line with protein/mRNA expression data in CRC on these transcription factors that have been published within the last five years [8], [9], [10], [13]. To obtain further validation of our mRNA expression data, we wanted to compare the mRNA data with the protein expression directly. This was possible on the remaining FFPE material of the same tissue block. We focused our validation study on the protein level on SNAI1 because of the statistical significant association with decreased CDH1 expression. Using a previously published [18] and commercially available Snail1-antibody, we could validate the mRNA data of SNAI1. We also observed a trend between nuclear Snail1 protein expression and decreased CDH1 protein (E-cadherin) expression. It is important to stress that the Snail1 immunohistochemistry displayed a nuclear localization reflecting its function as transcription factor.

According to our data, SNAI1, but not TWIST1, seems to contribute significantly to the down-regulation of E-cadherin in benign colorectal adenomas, in which decreased E-cadherin levels have already been described [24], [25], [26], [27]. Does that mean that EMT processes driving metastasis are already active in a benign premalignant condition? In transgenic mouse models of cancer it could indeed be demonstrated that tumor cells disseminate in morphologically benign appearing hyperplastic lesions [28], [29]. Subsequent molecular analyses of the hyperplastic tissue in the breast cancer mouse model revealed a strong up-regulation of TWIST1 as well as of proteolytic enzymes suggesting EMT as an underlying mechanism for early tumor cell spread [29]. In human cancer however, only very few data are available indicating that tumor cell dissemination could take place in morphologically non-invasive tumors. There are convincing data demonstrating that patients with DCIS already harbour cytokeratin-positive DTC in their bone marrow [29], [30]. In the case of colorectal cancer progression, Steinert et al. [31] reported about cytokeratin-positive as well as EpCAM-positive bone marrow DTC in a small series of patients with colorectal adenomas. These data by Steinert et al were controversially discussed and technical concerns were raised [32]. Therefore, a note of caution is required; especially since no independent and confirmatory data for the prevalence of bone marrow DTCs in adenoma patients are available. However, Pantel et al. just recently demonstrated using the FDA cleared CellSearch system that EpCAM positive circulating tumor cells (CTCs) can be already detected in the peripheral blood of benign colon diseases including adenomas [33]. But regarding the low annual transition rate of 2.6–5.6% to CRC even for advanced adenomas and the curative effect of simple endoscopic polypectomy [34], [35], [36], clinically relevant occult tumor cell dissemination seems very unlikely at this stage of CRC progression. Thus, the detected “signature” suggestive of EMT observed in a fraction of colorectal adenomas could rather reflect aberrant gene expression in the setting of tissue reorganization and expansion of less differentiated cells during adenoma growth [37], [38]. Besides, to confirm EMT several other markers would need to be analysed. This would be important, especially because (i) all adenoma cells still displayed a typical epithelial morphology, and (ii) the E-cadherin expression was admittedly reduced compared to normal mucosa, but still preserved in all cases classified as “reduced expression”.

A potentially contradictory result of our study was the noted co-expression of CDH1/E-cadherin and CDH2. This observation is however consistent with findings by Rosivatz et al [23], who detected CDH1/CDH2 co-expression in 33 of 80 (41%) colorectal carcinomas as well as 4 of 6 (66%) invasive colorectal carcinoma cell-lines. They interpreted the co-expression as a sign for N-cadherin's ability to suppress the function of E-cadherin. This hypothesis was supported by Nieman et al [17], who observed an increase in motility and invasion in previously non-invasive, E-cadherin positive breast cancer cell-lines upon forced co-expression of N-cadherin. The forced co-expression of E-cadherin in invasive, N-cadherin positive cells did not suppress their ability to invade and migrate, either. However, further studies are clearly necessary to investigate whether “real” EMT takes place by a more extensive profiling of EMT-markers.

In conclusion, our hypothesis generating study revealed SNAI1 expression as well as combined SNAI1/TWIST expression to be associated with decreased expression of CDH1 in colorectal adenomas. Whether the expression of the EMT transcription factors has an influence on the malignant potential of the colorectal adenomas was not addressed in our study. However, it is of interest that a recent transcriptome profiling study comprising over 320 CRC revealed an EMT-signature as the dominant pattern of intrinsic gene expression. This EMT-signature was tightly correlated with shortened relapse-free survival. Major components of the signature were up-regulated TWIST and down-regulated CDH1 [10]. Therefore, further investigation might be beneficial to check the use of TWIST1 and SNAI1 as markers for high-risk colorectal adenomas.
